# Garlic (*Allium sativum*) based interplanting alters the heavy metals absorption and bacterial diversity in neighboring plants

**DOI:** 10.1038/s41598-021-85269-4

**Published:** 2021-03-12

**Authors:** Javed Hussain, Xiao Wei, Luo Xue-Gang, Syed Rehmat Ullah Shah, Muhammad Aslam, Imtiaz Ahmed, Shaikh Abdullah, Asma Babar, Ali Murad Jakhar, Toquier Azam

**Affiliations:** 1 School of Life Science and Engineering, Southwest University of Science and Technology, 59# Qinglong Road, Fucheng District, Mianyang, 621010 Sichuan China; 2 Lasbela University of Agriculture, Water and Marine Sciences, Uthal, Balochistan 90150 Pakistan

**Keywords:** Plant sciences, Environmental sciences, Natural hazards, Planetary science

## Abstract

Heavy metals are naturally occurring elements that have a high atomic weight and let out in the environment by agriculture, industry, mining and therapeutic expertise and thrilling amassing of these elements pollutes the environment. In this study we have investigated the potential of garlic interplanting in promoting hyper accumulation and absorption of heavy metals to provide a basis for phytoremediation of polluted land. Monoculture and inter-plantation of garlic were conducted to investigate the absorption of cadmium and lead contamination in the land. A group of experiments with single planting (monoculture) of *Lolium perenne*, *Conyza canadensis* and *Pteris vittata* as accumulators were used. The results have shown that garlic has a potential as a hyper accumulate and absorb heavy metals. It was found that the accumulation of Cd and Pb was much higher with inter-planting. Garlic boosts up the absorption of heavy metals in *Lolium perenne* of Cd 66% and Pb 44% respectively. The Inter-planting of garlic with *Pteris vittata* promotes the Cd 26% and Pb 15%. While the maximum accumulation of Lead 87% and Cadmium 77% occurred in *Conyza canadensis* herb plant. The bacterial diversity in the soil was analyzed for each experimental soil and was found that the *Proteobacteria, Acidobacteria, Actinobacteria, Firmicutes,* and *Planctomycetes* were commonly abundant in both single planting (monoculture) of ryegrass and interplanting ryegrass with garlic habitats. Variances were observed in the bacterial floral composition of single (monoculture) and intercropping (interplant) soils. Relative abundance of bacterial taxa revealed that the proportion of *Proteobacteria, Acidobacteria*, and *Actinobacteria* in the inter-planting group was slightly higher, while *Firmicutes* and *Planctomycetes* were low. This study provides the evidence to control the heavy metals contaminated soils with weed species. Growth promotion and heavy metal uptake of neighboring plants proved the specific plant-plant and plant-microbial associations with garlic plants. This inter-planting strategy can be used to improve heavy metal absorption.

## Introduction

Heavy metals are naturally occurring elements that have a high atomic weight and a density at least five times greater than that of water^1^. Heavy metals released into the environment by agriculture, industry, mining and therapeutic expertise. Substantial release of these elements pollutes the environment^2^. Heavy metal toxicity has proven to be a major threat and there are several health risks associated with it. The toxic effects of these metals, even though they do not have any biological role, remain present in some or the other form harmful for the human body^3^. Agricultural practices have been one of the main sources of heavy metals in soil such as lead, chromium, arsenic, zinc, cadmium, copper and nickel^4^.

Even at low concentration some of the heavy metals in the soil solutions are very toxic such as lead (Pb), cadmium (Cd), which do not have any role in the plant metabolism thus can be eliminated from the environment through various techniques. These techniques include excavation, solidification, burial, soil washing, use of microorganisms and phytoremediation. Various technologies have their both beneficial and harmful effects, however phytoremediation is found to be cost effective and more environment friendly technology^5^. A serious problem of contamination of Cd is caused by agricultural land through irrigation of paddy fields from mining activities, fallout dust from metal refineries on field and application of Cd-rich fertilizers, causing serious threats to human’s health and environment, unlike some other nonessential elements^6,7^. *Pteris vittata* transport and accumulate Cd in the shoots such as stems and leafs, delocalizing it in the less bioactive tissues of the frond^8^. Pb^2+^ has long lasting effects once the soil is contaminated and it remains in the soil due to its non-biodegradable nature and was found to be highly toxic to human beings when present in high amounts. High contaminated soil seldom returns to normal without remediation. Lead is known to be toxic to plants, animals and microorganisms in the environment and it can cause brain damage, retardation and pose serious human health problems if it exists in the environment as an insoluble form^9,10^. Soil remediation techniques mainly include physical remediation, chemical remediation and bioremediation. Phytoremediation is a green technology with good public reception and is a better option to clean up heavy metal-contaminated sites. Accumulators are the plants which absorb heavy metals and hyperaccumulators absorb more than other plants^11^. Hyperaccumulators have the ability to grow in metalliferous soils^12^. Normally the accumulators which grow in heavy metals contaminated soil but these accumulators have no higher concentration of heavy metals than the Standard of State Agriculture Production Safe Quality GB 2762-2005^13^. Phytoremediation process can be enhanced by screening plants for hyper accumulation of heavy metals and development in molecular studies can improve the efficiency of accumulators^14^. It is well known that plants such as *P. vittata*, *Lolium perenne* and *Conyza canadensis* are hyper-accumulators that are more efficient to remove heavy metals from polluted soils^15–17^. Under moderate to severe contamination conditions of soil, inter-planting patterns could be an efficient way to enrich absorption of heavy metals. For remediation of agricultural soil, especially for the restoration of cultivated soil, ensuring minimum impact on agricultural production is considered for inter-planting crops and hyperaccumulators. Until a few investigations have been revealed to show results, including the effects of Cd_2_ concentration on the garlic (*A. sativum*) root, bulb and shoot accumulation. However, the plants transported only a small amount of Cd to their bulbs and shoots^18^. The differential uptake of Pb in root and shoot is may be due to the uptake of lead from the aqueous phase of the soil by the natural ion uptake mechanisms of the roots^19^. The intercropping of garlic with other plants is helpful to absorb the heavy metal contents from contaminated soils. Further, the inter-planting application protects other plants companions by decreasing the heavy metal content from the rhizosphere^20^. Few studies have been conducted with inter-planting to promote or weaken hyperaccumulate absorption of heavy metals^21^. Hyper-metal-accumulating plants alone for phytoextraction of heavy metals may not be so efficient in some cases due to the inability of a single hyper-accumulator to remove all contaminants from the soil due to their slow growth and low biomass production. Garlic is known to be for its ability to resist biotic and abiotic environmental stresses like bacterial, viral and oxidative stresses^22^ and have a potential to persist against some heavy metals such as cadmium. Garlic as a spice plant was chosen as an intercrop plant with hyper-accumulator for its effect on soil microbial activities of heavy metals in soil^23,24^. The inter-planting is concerned, this arena has attracted the attention of soil scientists to remediate the soil contamination of heavy metals^25,26^. The inter-planting has long been in practice in Chinese agriculture field, for efficient light interception, increased soil fertility and role in nitrogen fixation in soil^27,28^. Inter-planting has some promising results to hyper-accumulate the heavy metal content from soil resulting in the reduction of the heavy metal content in soil. This practice paves the way to safe agriculture which in turn beneficial for human consumption as well as betterment of the ecosystem. Inter-planting is pivotal to enhance crop yield, phytoextraction of heavy metals, augment soil enzymatic activities, microbial diversity, alleviation of the soil microbial activity and improvement of the physico-chemical properties of soil by reducing the heavy metal content in contaminated soil^29–31^. Plants harbor a number of microbial communities in the rhizosphere ecosystem. The positive interactions of these microbes increase rhizosphere soil fertility while some of the microbial biota competes with the plants for nutrients and in addition to the other resources. Previously, some of the studies focused on microbial properties of the rhizosphere of *Lolium perenne* for Cd and Pb contaminated soils^32,33^. In this study three plant species (*Lolium perenne*, *Conyza canadensis* and *P. vittata*) were selected to investigate monoculture and intercropping with garlic in different combinations using a greenhouse designed experiment to evaluate the comparative analysis of monoculture and intercropping for the heavy metal remedy of contaminated soil and the changes in diversity of microbial communities within contaminated soil. Therefore, it is hypothesized that intercropping of *Lolium perenne* with garlic can increase the microbial diversity within soil as a result of which the enzymatic activity of soil may increase several fold depending upon density of intercropping and microbial interactions within the contaminated soil. The main objective of this research was to measure the potential of garlic in interplanting with *P. vittata*, *Conyza canadensis* and *Lolium perenne* to enhance accumulation of heavy metals to detoxify lead and cadmium from the contaminated soil.

## Results and discussion

### Comparison of biomass

**Figure 1:**
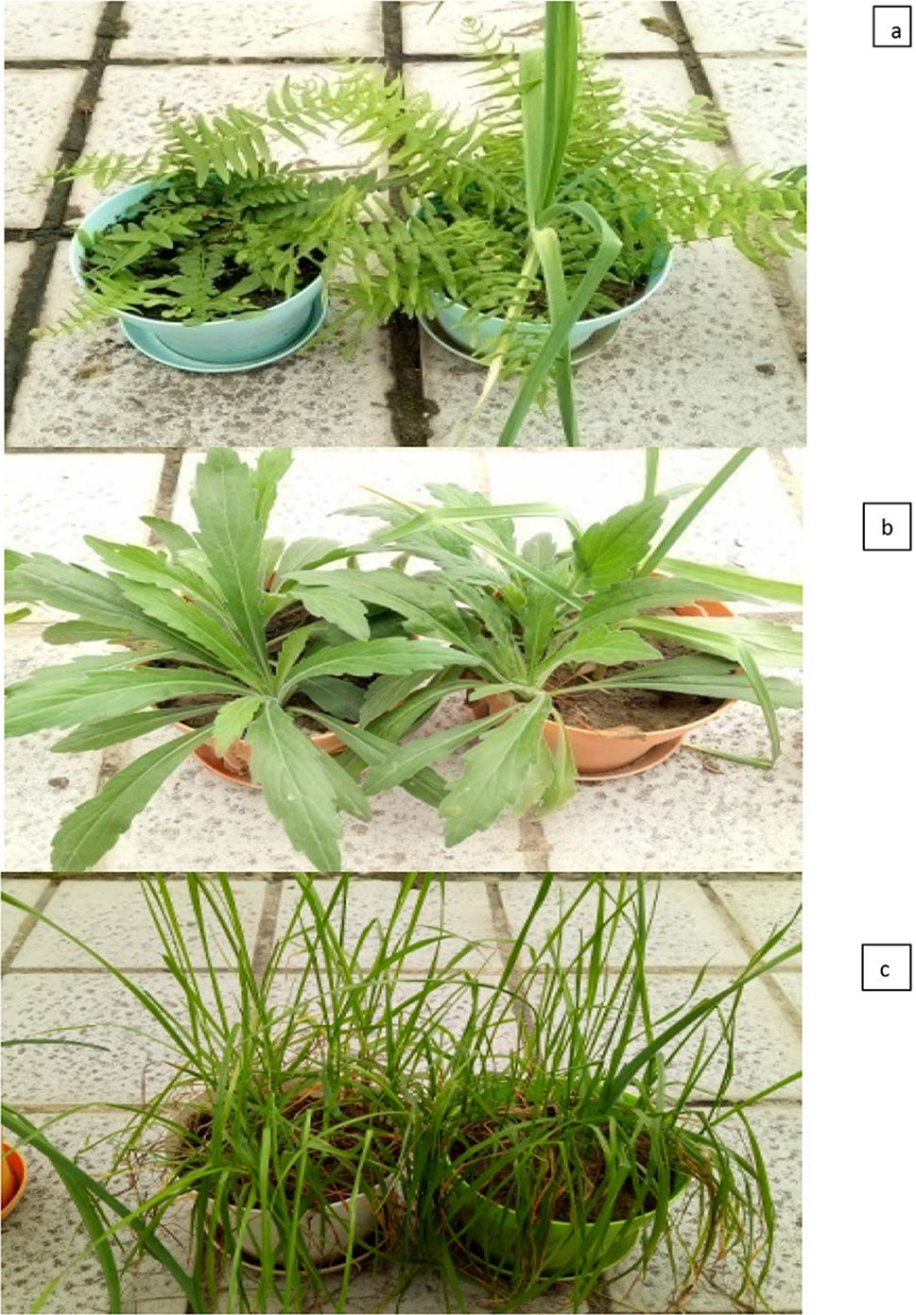
Single planting and inter-planting treatments of Garlic with *P. vitatta* (**a**), *Conyza canadensis* (**b**) and *Lolium perenne* (**c**).

Analyses of dried plant biomass of *Lolium perenne*, *P. vittata*, *Conyza canadensis* have been grown for 60 days on heavy metal treated soil under monoculture and the inter-planting systems. Dry biomass of *P. vittata*, *Conyza canadensis* and *Lolium perenne* in inter-planting with garlic growth was increased as compared to monoculture. The physiological responses in the inter-planting system helps the plants to overcome the heavy metal stress in contaminated soil, plants in monoculture possess lesser shoots, roots biomass and heavy metal accumulation ability. Whereas, inter-planting with garlic showed higher level shoots, roots biomass and can acquire an assessment tools and a valuable information for hyper-accumulator of heavy metal tolerance plants and help to remediate heavy metals^34^ (Fig. 1). Usually in the inter-planting pattern the plants compete for their essential resources like space, growth and supply of essential nutrients that is responsible to inhibit the plant growth of any of the competitors^26,35^. The stimulation of growth by growing the plants together in presence of garlic indicates a largely beneficial effect of inter-planting. Previously, the inter-planting has been successfully adapted to increase the hyper-accumulation by *P. vittata* the plant biomass increased significantly^26,29^. Subsequently the total Cadmium (Cd) and lead (Pb) content of inter-planting and monoculture treatments as seen in (Table 1) for 60 days of soil remediation depends on the biomass and absorption capacity of individual plants. *P. vittata, Conyza canadensis, Lolium perenne* in monoculture were significantly lesser (p < 0.05) than that of inter-planting with garlic. Furthermore, the garlic was helpful in Pb and Cd extraction from the heavy metal contaminated soil. Whereas, the total Cd accumulation in monocultures *P. vittata* and *Conyza canadensis* was significantly decreased except in *Lolium perenne.* as compared with that in inter-planting. The Pb accumulation value was seen lesser in monoculture. *P. vittata*, *Conyza canadensis* and *Lolium perenne* with garlic increased, as compared to the single culture of *P. vittata*, *Conyza canadensis* and *Lolium perenne*, *P. vittata*, *Conyza canadensis* and *Lolium perenne* have poor accumulation capacity for cadmium and lead in single culture (Table 2). The intercropping of garlic with *P. vittata*, *Conyza canadensis*, and *Lolium perenne* resulted in higher underground accumulation and lower Pb and Cd above-ground accumulation, indicating that the above-mentioned inter-plant cultivation will be a good choice. The plant extracts of Cadmium (Cd) and lead (Pb) in the monoculture treatment of *P. vittata*, *Conyza canadensis* and *Lolium perenne*, it is best to show complementarity between hyper-accumulators and heavy metal tolerance through intercropping. Inter-planting effectively increases the complementary high accumulation of heavy metal like cadmium and lead-resistant species, and their wide application to remediate the heavy metal content in the soil. In present study inter-planting of garlic with three plants, was found to be very effective to remediate the soil status contaminated with Cd and Pb, instead of using three plant species in monoculture. However, the long term application of field trials is underway to mark the feasibility of three inter-plant species in the remedy of contaminated soils.

**Table 1:** Mean values for biomasses of aerial part and underground part of single plant and interplant garlic with *P. vittata, Conyza canadensis and Lolium perenne*.

Plants	N	Single planting		Inter-planting	
		Aerial part (shoot) (mg/kg)	Underground part (root) (mg/kg)	Aerial part (shoot) (mg/kg)	Underground part (root) (mg/kg)
*P. vittata*	3	0.7 ± 0.3	0.3 ± 0.2	1.4 ± 0.3	0.5 ± 0.1
*Conyza canadensis*	3	2.1 ± 0.2	1.5 ± 0.1	2.9 ± 0.4	1.7 ± 0.1
*Lolium perenne*	3	1.8 ± 0.5	0.9 ± 0.4	1.8 ± 0.2	1.3 ± 0.4

*N* number of plants sampled.

**Table 2:** Mean values for cadmium and lead content comparisons in single planting and interplanting with garlic.

Plants	N	Single planting				Inter-planting			
		Shoots		Roots		Shoots		Roots	
		Cd (mg/kg)	Pb (mg/kg)	Cd (mg/kg)	Pb (mg/kg)	Cd (mg/kg)	Pb (mg/kg)	Cd (mg/kg)	Pb (mg/kg)
*P.vittata*	3	0.096 ± 0.013	2.545 ± 0.067	1.902 ± 0.043	11.964 ± 1.25	0.098 ± 0.019	2.685 ± 0.015	4.796 ± 0.051	12.335 ± 0.322
*Conyza Canadensis*	3	0.476 ± 0.063	1.308 ± 0.043	0.784 ± 0.117	8.517 ± 0.136	0.600 ± 0.071	2.044 ± 0.175	1.020 ± 0.069	9.558 ± 0.297
*Lolium Perenne*	3	0.315 ± 0.024	3.184 ± 0.157	1.168 ± 0.060	10.996 ± 1.439	0.298 ± 0.029	5.307 ± 0.167	1.338 ± 0.039	13.282 ± 0.558

*N* number of plants sampled.

### Lead and cadmium accumulation

The Cd and Pb quantity of shoots and roots in the -metal hyper-accumulators in single and inter-planting with garlic are presented in (Table 2) The concentration of Cd in the roots of *P. vittata* were much higher in inter-planting than single planting, while Cd concentration in shoots were not significantly different in both single and inter-planting setups. Similarly Cd concentrations in the roots of *Lolium perenne* were higher in inter-planting when compared with single planting. However Cd concentration in shoots remained lower than in the roots. *Conyza canadensis* when grown in monoculture showed that the concentration of Cd in shoots and roots was slightly lower than the inter-planting concentration. A rising trend in concentration of Pb in the roots of *P. vittata*, *Conyza canadensis* and *Lolium perenne* in Inter-planting were observed when compared with single planting (Table 2), while Pb concentration (mean values) in shoots of all plants were minutely increased when compared with single planting setup.

Higher amount of Pb in *Lolium perenne* roots in inter-planting with garlic suggests better uptake and translocation and mobilization within soil in the co-cultivation with garlic. Previously, not much is known about uptake of heavy metals in intercropping with garlic and this study interestingly shown that, association of garlic with specific species can alter the heavy metal uptake in neighboring plants indicating that the above-mentioned inter-plant cultivation will be a good choice for phytoremediation of soil. The plant extracts of Cd and Pb in the monoculture treatment of *Conyza canadensis* and *Lolium perenne*, it is best to show complementarily between hyper-accumulators and heavy metal tolerance through intercropping. This strategy to remove heavy metals Cd and Pb on a contaminated soil, can be optimized by inter-planting of *P. vittata*, *Conyza canadensis* and *Lolium perenne* with garlic^32^. Other studies also confirm the similar results in the interplant system, which effectively used *P. vittata*, *Conyza canadensis* and *Lolium perenne* to remove cadmium and lead from the soil^43^. With this study, it is recommended that *P. vittata*, *Conyza canadensis*, and *Lolium perenne* inter-planted with garlic provides the flexible and effective phytoremediation to contaminated soil with Cd and Pb. Therefore, Inter-plantation of multiple plants can improve the phytoremediation of heavy metals so very effectively and it is less time consuming process to remediate soils through plant remediation^36^. The results (Table 3) describe concentration of Cd and Pb in the edible part of garlic in an inter-planted setup with *Conyza canadensis* and *Lolium perenne*. The concentration in the edible parts is much lower than roots. Previously in a hydroponically grown garlic experiment maximum concentration of heavy metals was observed in roots as compared to bulbs and shoots. Garlic bulbs are reported to contain the lowest concentration of heavy metals as compared to other *Allium* species^37^ and our study suggested it is not only the species difference but also the association of neighboring plants that affects the uptake of heavy metal in garlic.

**Table 3:** Mean values of cadmium and lead content garlic edible part (without roots).

Treatments	N	Cadmium content (mg/kg)	Lead content (mg/kg)
Garlic with *P. vittata*	3	0.298 ± 0.034	1.382 ± 0.120
Garlic with *Conyza canadensis*	3	0.093 ± 0.012	0.887 ± 0.114
Garlic with *Lolium perenne*	3	0.107 ± 0.073	2.081 ± 0.154

*N* number of sampled plants.

### Microbial richness and diversity

Through MiSeq analysis of the V3–V4 region of bacterial 16S ribosomal RNA genes, a total of 223,933 sequences of 16S rRNA were acquired from the six soil samples and the rearranged sequence reads number per samples ranged from 31,207 to 41,412. The optimized read sequence numbers were uneven. Each sample was randomly reduced to the size using the MOTHUR basing to the smallest read number (24,712). The genetic distances of 3% OTUs were identified and a total of 11,930 OTUs were acquired from the samples ranging 5848 from the interplant *Lolium perenne* with garlic, 6082 single planting *Lolium perenne*, respectively covering 99% in each of the samples.

The randomly extracted sequences were clustered to OTUs with an average of 2819 ± 273 OTUs per sample (min = 2272, max = 3275; “Supporting Information” (Table 4)). ACE richness estimator mean in single plant ryegrass soil was slightly higher than interplant ryegrass with garlic. Moreover the same value was obtained from the Chao richness estimator. The richness and diversity estimators (ACE, Chao, Shannon) of single plant ryegrass soils were slightly higher than those of the interplant ryegrass with garlic. This decrease in the richness and diversity of microbial communities may be due to the antimicrobial effects of Garlic in inter-planting setup^38,39^. Garlic has a large spectrum of activities such as generating significant antioxidant and antimicrobial effects^40,41^. Biotic and abiotic environmental stress is well known for its characteristic resistance^18^ identified a variety of microbial communities in similar studies.

**Table 4:** Bacterial diversity and richness in soil of single plant and interplant ryegrass with garlic Cluster distance (0.03).

Sample\estimators	ACE	Chao	Coverage	Shannon	Simpson	Sobs
*Lolium perenne*	2241.781	2231.07	0.989943	6.491043	0.003701	2075
*Lolium perenne* garlic	2260.26	2291.342	0.986906	6.374229	0.004486	2012
*Lolium perenne*	2229.341	2219.101	0.987077	6.407915	0.004194	2022
*Lolium perenne* garlic	2187.282	2153.976	0.986683	6.269381	0.006023	1953
*Lolium perenne*	2198.869	2157.509	0.985918	6.448085	0.004118	1985
*Lolium perenne* garlic	2184.903	2172.441	0.983058	6.312332	0.00432	1883
					Total	11,930

### Taxonomic composition of bacterial communities

**Figure 2:**
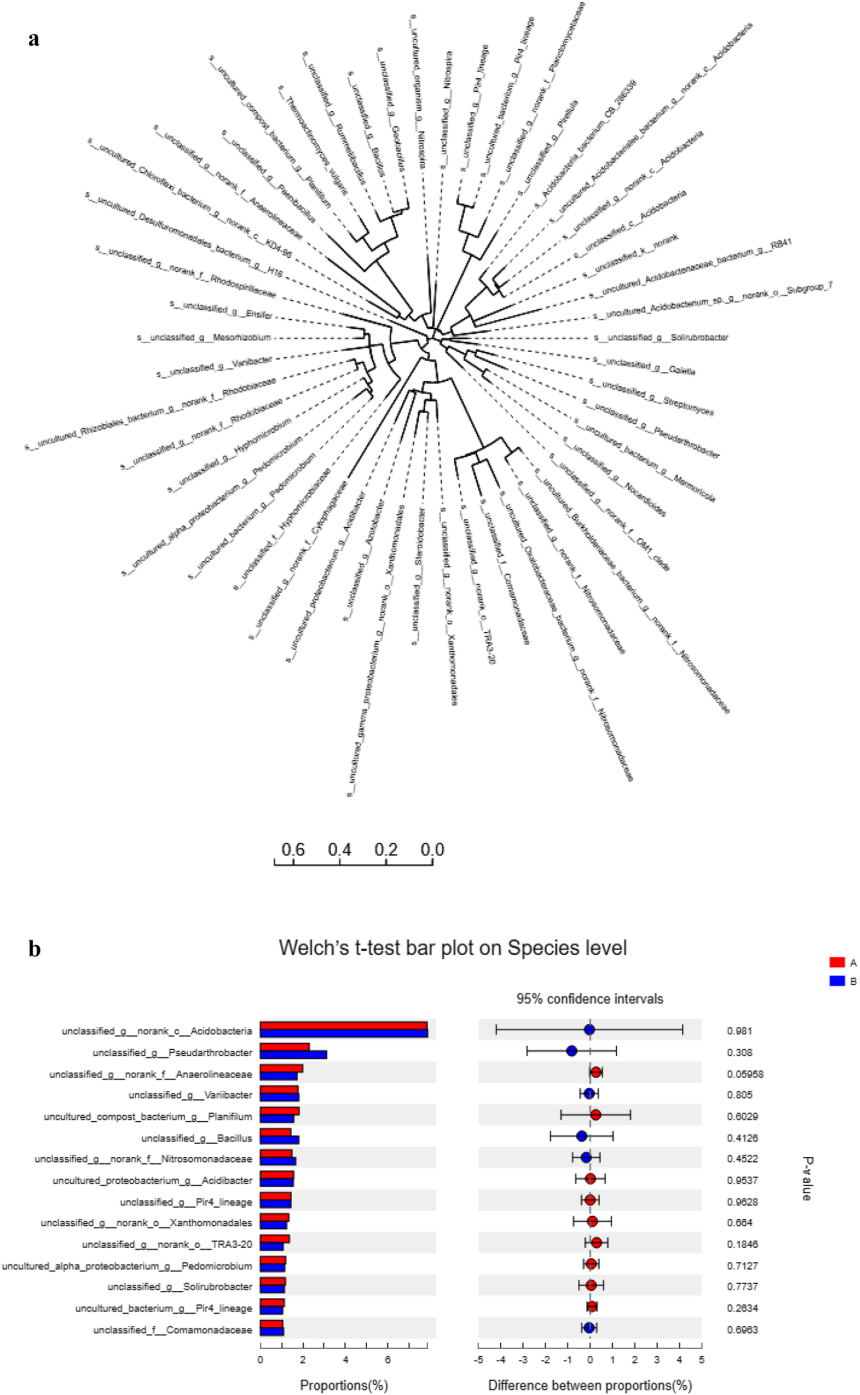
(**a**) Each branch of phylogenetic tree represents a species and the length of the branch is the evolutionary distance between the two species, that is the degree of difference in the species. (**b**) Relative abundance of the bacterial species in single (A) and interplant cultures (B). The vertical axis represents the species name at a certain classification level, and each bar corresponding to the species represents the average relative abundance of the species in each sample group, and the different colors indicate different group in the middle area is the difference between the abundance percentages of the two groups in the set confidence interval. The color of the dot is displayed as the group color with a large abundance of species and the type I interval on the dot is the upper and lower limits of the difference value. The rightmost side is the P value, *0.01 < P ≤ 0.05**0.001 < P ≤ 0.01***P ≤ 0.001.

**Figure 3:**
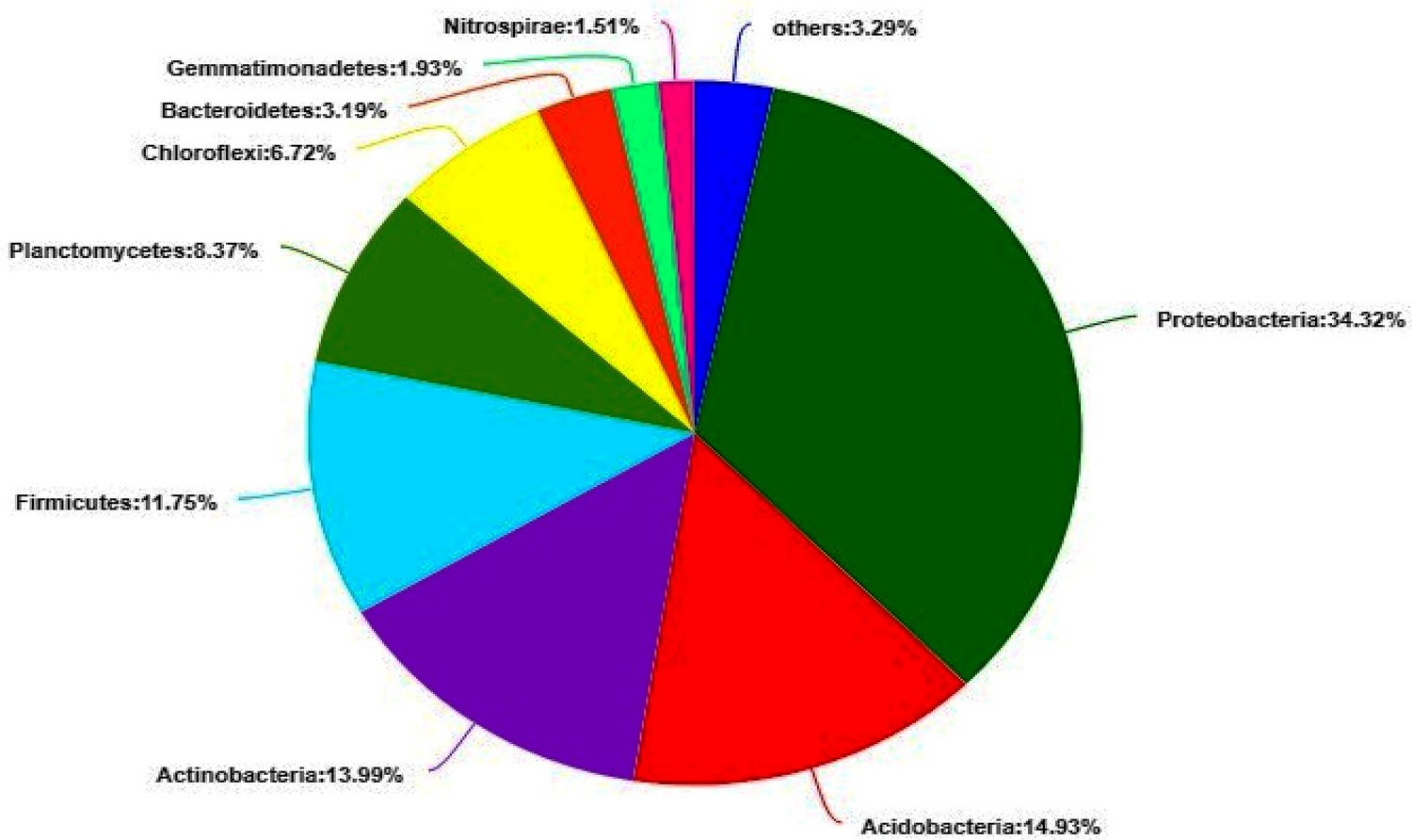
Common phyla relative abundance of soil bacterial community shown as percentage of pooled data from single and interplant treatments.

Distinct sequences from each OTU annotated with SILVA databases revealed taxonomic classification of different bacterial community abundance at phylogenetic levels from the each soil sample. At phylum level, the differences among the two sets of samples were found not significant (Table 5).The treatments have affected more at individual genera linking them to treatment specificity (see Supplementary Fig. S1 online). Total 31 phyla were found in all soil samples (Fig. 2a). The dominant bacterial phyla in each sample were the *Proteobacteria* 34.8%, *Acidobacteria* 14.9%, *Actinobacteria* 13.9%, *Firmicutes* 11.7%, *Planctomycetes* 8.3%, *Chloroflexi* 6.7%, *Bacteroidetes* 3.1%, *Gemmatimonadetes* 1.9% and *Nitrospirace* 1.5%, respectively (Fig. 3). Whereas primary bacterial phyla were *Proteobacteria followed by Acidobacteria* and *Actinobacteria* same as in most of the rhizosphere bacterial phyla^39,42,43^. Rest of the abundant phyla, *Firmicutes* and *Bacteroidetes* in the order 4 and 7, respectively are shown differently in abundance order from previous study on the rhizosphere microbiome of different plant taxa. Phyla, including *Armatimonadetes*, *Firmicutes*, *Latescibacteria*, *Verrucomiocrobia* and other bacterial phyla sequences having very low proportions (< 1%). Bacterial species with low abundance could be the slow growing bacterial species which showed more variation between single plant and interplanting (see Supplementary Fig. S1 online) as compared to abundant flora^39^.

**Table 5:** Difference in bacteria phyla between the analyzed groups of single planting and cultivated *Lolium Perenne* with garlic interplanting.

Phylum Name	N	Single planting-mean (%)	SD (%)	Interplanting mean (%)	SD (%)	P-value
*Proteobacteria*	3	33.63	0.7159	35.8	4.344	1
*Acidobacteria*	3	14.94	0.4736	15.31	4.241	1
*Actinobacteria*	3	12.95	1.965	14.96	1.445	0.7728
*Firmicutes*	3	12.46	2.89	9.959	0.5379	0.1489
*Planctomycetes*	3	8.758	0.3376	7.885	0.4378	0.1489
*Chloroflexi*	3	6.974	0.3859	6.414	0.3891	0.1489
*Bacteroidetes*	3	3.456	0.2836	3.09	0.0887	0.1489
*Gemmatimonadetes*	3	1.93	0.1152	1.981	0.7983	1
*Nitrospirae*	3	1.43	0.1527	1.544	0.3119	0.7728

The interactive analysis results, Mean is the mean, Sd is the standard deviation; P value is the false positive probability value.

*N* number of plants sampled.

Comparing the single and Interplanting *Lollium perenne* with garlic (Table 5), the abundance of *Proteobacteria*, *Acidobacteria* and *Actinobacteria* is increased (mean values) in interplanting, while percentage of *Firmicutes* and *Planctomycetes* is decreased. Other phyla including *Chloroflexi*, *Bacteroidetes*, *Gemmatimonadetes*, *Nitrospirae*, *Latescibacteria*, *Cyanobacteria*, *Armatimonadetes*, *Tectomicrobia*, *Chlorobi* were not changed. Low variation at phyla level is not surprising due to overcompensation of species within each phyla from monoculture or interplanting (Fig. 2b). Physicochemical properties of soil in interplant may affect indirectly changes through plant-mediated in soil microbial community, improves the bacterial diversity and richness^38^, Rhizobia, potassium-solubilizing bacteria may be the nutrients released by the agroforestry trees related to increased abundances of bacterium^44^. The heavy metals concentrations directly affect the microbial diversity and limit the growth of microbial species usually present on the soils^45^, reported the negative correlation of the abundance of *Gemmatimonadetes, Nitrospirae,* and *Planctomycetes* microbial communities with total Pb. They reported that the heavy metals (Pb) can influence the microbial community composition and physiological properties of soil^39^.

### Shared and distinct bacterial OTUs

**Figure 4:**
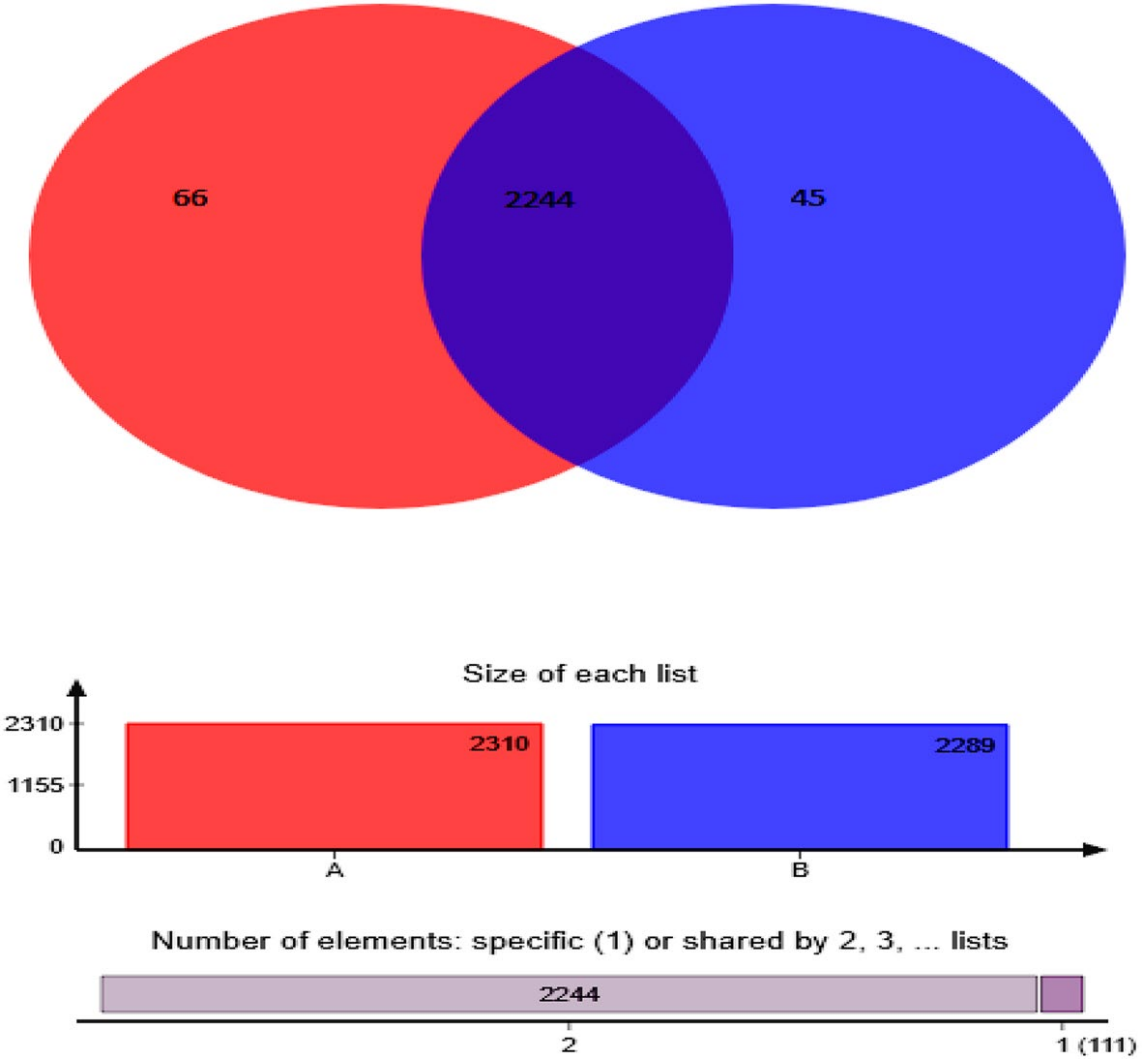
Distribution of bacterial OTUs in all soil samples showing in Venn diagram for Single plant is represented by (A) and interplant by (B). Overlapping area in Venn diagram represents the shared bacterial OTUs between single plant and interplant treatments.

**Figure 5:**
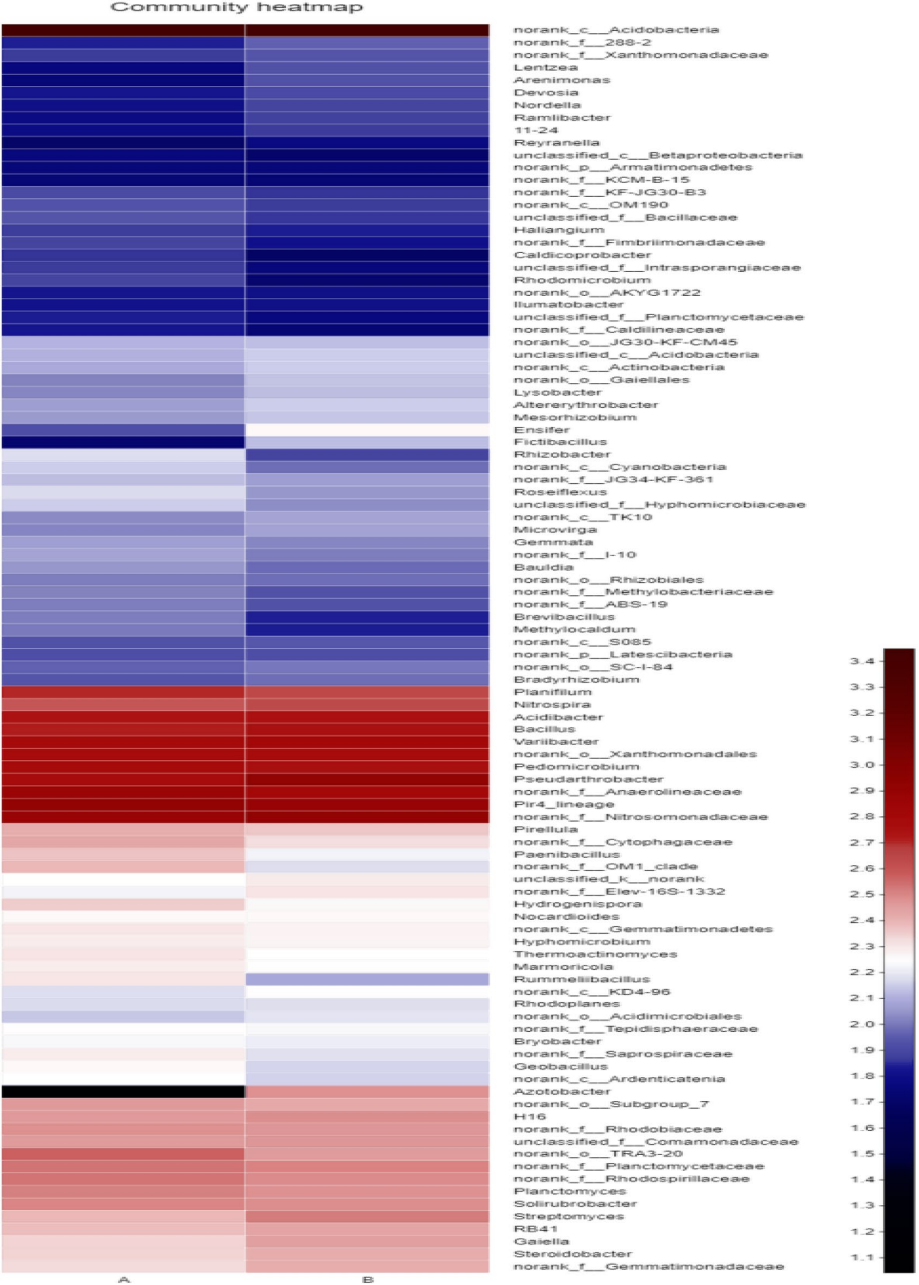
Hierarchical cluster analysis of 100 main OTUs bacterial communities were determined by genus. Scale indicates the relative abundance of each OTU read color intensity. Clustered based on complete linkage method were applied for samples communities.

Total 2354 OTUs were observed in soil samples, whereas 2244 OTUs were common to all soil as shown in the Venn diagram (Fig. 4). Distribution of sequences showed that 66 OTUs in single plant and 45 OTUs in interplant were detected solely in those environments. The genus level microbial community based on hierarchically clustered heatmap analysis was utilized to distinguish the dissimilar arrangement of the microbial community. Single planting *Lolium perenne* represents (A) and (B) interplant *Lolium perenne* with garlic group indicated slight differences of microbial community composition between the two group treatments (Fig. 5).

**Figure 6:**
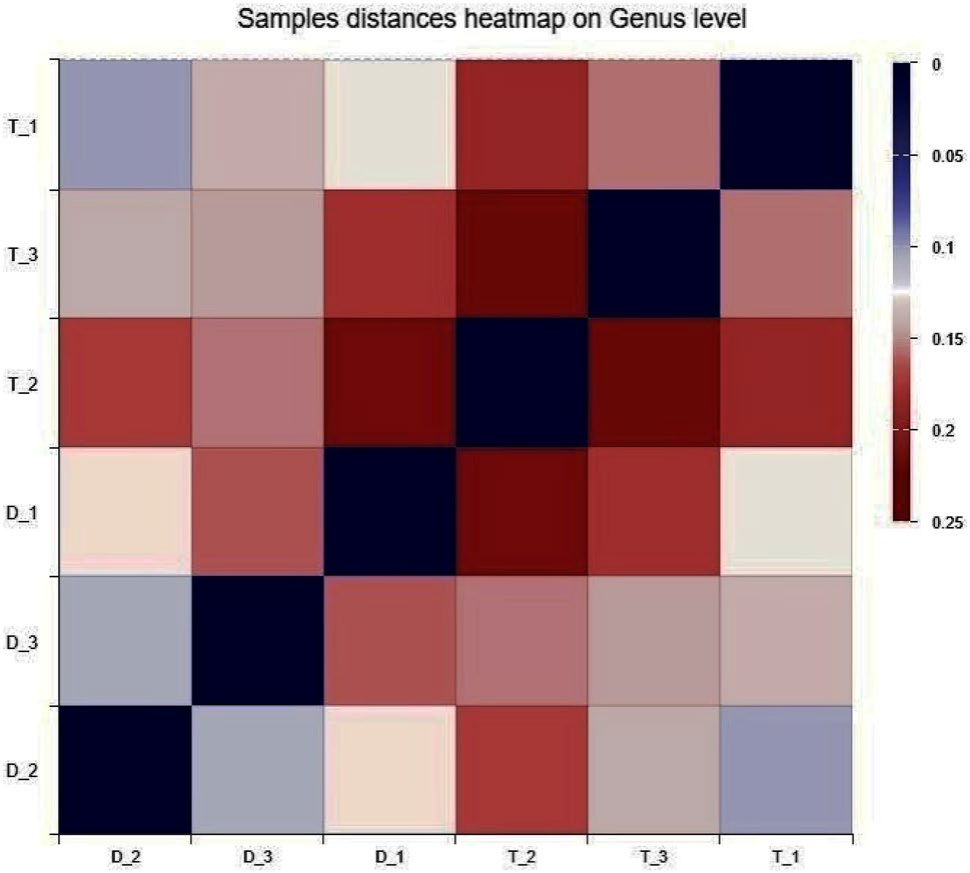
Distance dissimilarity heatmap on Genus level of Bray–Curtis dissimilarity matrix of Single and interplant groups (T_1, T_2, T_3 represents single Treatments, while D_1, D_2 and D_3 represent interplant treatments). According to the standard protocols by Majorbio Bio Pharm Technology Co. Ltd. (Shanghai, China), using Sanger software (https://www.i-sanger.com/).

The dissimilarity matrix indicates the unlike values among the single and interplant treatments (Fig. 6). Planting patterns and different plant species suggested that soil bacterial community structure and distance between the community structures is shown as a dissimilarity matrix. Observing the biodiversity of the resident community, the effect of taxonomic richness mainly due to functional dissimilarity remained significant. Host plant species determine their rhizosphere microbial species^43^. This can be a one-way or mutual association between the bacteria and host plant species. Selection of the bacterial community depends on the inoculum present in that soil^46,47^. Variation in species shows functional differences of plant species within each rhizosphere inform of root exudation or signaling.

## Conclusion

In this study we found that intercropping of garlic with *P. vittata*, horseweed, and perennial ryegrass can effectively enhance the absorption of cadmium and lead from contaminated soils, as compared to that of monoculture planting. The intercropping of garlic with *P. vittata*, horseweed, and perennial ryegrass resulted in higher underground (in roots) accumulation and lower above-ground (in shoots) accumulation of Pb and Cd indicating that proposed inter-plant cultivation potentially be a good phytoremediation choice for the amelioration of soil chemistry. Furthermore inter-planting system can helps the plants to overcome the heavy metal stress, however, may not affect plant biomass significantly when compared with monoculture, further investigations are suggested in this context.

The richness and diversity of soil microbial community may be inhibited due to the antimicrobial effects of Garlic in inter- planting setups, however, soil enzyme activity should be considered in further investigations, as soil enzyme activity is a major contributing factor to overall biological characteristics of contaminated soil. Despite that, Inter-planting accumulator plants *P. vittata*, horseweed herb and perennial ryegrass with garlic can have pivotal role in the interaction between soil and microbes within the ecosystem to remediate the heavy metal contaminated soils, but this needs to be verified.

This study indicates that inter-planting with garlic can achieve higher extraction rates of heavy metal in plants, compared with single plant cultivation. Furthermore, inter-planting strategy can have its wide uses to restore ecological functioning, soil stabilization, restoration and reconstruction of soil contaminated with heavy metals.

## Materials and methods

### Site description and soil selection

All the experimental work was conducted in the months of January and March 2018 at Southwest University of Science and Technology located (104° 41′ 37″, 31° 32′ 18) at Mianyang Sichuan Province China. According to climate-data-org study the average temperature is 6–16 °C, and the annual average precipitation is 6–933 mm. The coldest month is December with low Precipitation commonly occurred, whereas warmest month is July with 26 °C. Soil samples were collected at 0–10 cm depth of soil layer from the University campus in the month of October, 2017 and immediately transported to the laboratory. Stone and other trash material were manually removed from the collected soil, and subsequently sieved through 2.0 mm mesh size. The soil samples were then divided into two aliquot sealed plastic bags one stored at 4 °C for physiochemical analysis. The soils to water ratio pH 8.12 were measured with pH meter (INESA- PHS-3C China), and electro conductivity 63.20 µs/cm were measured. Soil texture was determined as sand 14.5%, silt 29%, clay 56%, moisture content 26.65%, organic content was 15 g/kg, and carbon content was 130 g/kg. The heavy metals salts concentration used for the soil pot experiments were Lead nitrate (Pb) minimum concentrations were 100 mg/kg and maximum concentrations 300 mg/kg for each three replicates. Cadmium Chloride (Cd) concentration minimum 0.3 mg/kg and maximum0.9 mg/kg, respectively^14,48^. Soil mixture content were well mixed with 25% distilled water and kept in a dark place for 2 months metals and soil equilibrium state.

### Plant cultivation

The heavy metal treated soil of 1 kg, dry weight was placed in each pot (Ø = 12.5 cm, H = 15 cm). Plants of same size were selected and then transferred to experimental pots. The experimental work was performed in greenhouse conditions with 14 h daylight, the light intensity was 260–350 mmol/m^2^/_S_, the humidity was calculated as 50–65%, and the temperature was recorded in between 20 and 30 °C. According to the differences in growth morphology of three plant species, four plants were cultivated into two treatments monoculture, and intercropping with garlic. Two plants were used for each plant *Pteris vittata*, *Conyza canadensis* and 12 plants were used for *Lolium perenne*. Pot experiments were carried out with triplicate of each treatment. The treatments included monoculture and intercropping of garlic with a (*Pteris vittata*), dicot *Conyza canadensis* and a monocot *Lolium perenne*. Mature and healthy garlic seedlings were separated and kept in each plastic pot containing distilled water for one week in a greenhouse under controlled condition to get stabilized^22^*. P. vittata* spores were collected from the fertile leaves germinated in potting soil and irrigated with an interval of 4 weeks. When the germination occurred into the gametophyte stage, the plants were transferred to the pots^8^. *Lolium perenne* seeds were washed with distilled water and soaked with 10 ml of deionized water placed on filter paper at room temperature 22–24 °C under dark condition. Germinated plants were transplanted into contaminated soil. Meanwhile, all pots were watered using deionized water during cultivation to maintain the soil water content near to 60%. After 60 days of cultivation, soil from the same pots was sampled, then thoroughly homogenized. And the two portions of soil were taken, one portion of soil was air dried for determination of heavy metal content and the other remaining portion of soil was stored at − 80 °C for DNA extraction. The DNA analyses and microbial diversity was performed in triplicate and co-related with monoculture and intercropping^34^.

### Heavy metal analysis

Plants samples were washed with tap water followed with distilled water and dried with tissue paper. Plant leaves, stems and roots were separated. Plant tissues were dried at 80 °C for 72 h. Stainless steel electric grinder was used. The ground powder plant samples sieved through 2-mm mesh. Approximately 0.3 g of ground plant material (leaves and roots) were digested using microwave digester with hot-block solution containing 87% HNO_3_, 13% HClO_4_ for extraction. All the glassware were soaked overnight in 2 N HCl reagents with distilled–deionized water and washed according to standard methods before use in experiments. Stock solutions were prepared by appropriate dilutions of 1000 mg/L in a matrix which approximately match the acid concentration for the samples. Calibration curves were drawn from the 0, 5, 10, 20, 50, 100, 200, 500 and 1000 ppb standard solutions. The total metal concentrations were determined by Inductively Coupled Plasma (ICP-MS) Agilent Technologies 7700 series ICP-MS^16,49–51^.

### Microbial diversity analysis

Adhering plants material with soil particles was removed. Soil samples from each triplicate pot were mixed; single (monoculture) *Lolium perenne* and intercropping *Lolium perenne* with garlic were collected and immediately transported to the laboratory. The aliquots were stored at − 80 °C for DNA extraction and subsequently transported to Major-bio laboratory Shanghai China^52^.

### DNA extraction, PCR amplification

Microbial DNA was extracted from the collected soil samples using E.Z.N.A @ Soil DNA Kit (Omega Bio-tek, Norcross, GA, and US) following manufacturer’s protocols. The DNA concentration and purification were determined by NanoDrop 2000 UV–Vis Spectrophotometer (Thermo scientific Wilmington USA), and the DNA quality was checked by 1% agarose gel electrophoresis. The V3-V4 hypervariable regions of the bacteria 16S rRNA gene were amplified with primers 515F (5′-GTGCCAGCMGCCGCGG-3′) and primer 907R (5′-CCGTCAATTCMTTTRAGTTT-3′) by thermocycler PCR system (Gene Amp 9700, ABI, USA).The PCR reactions were conducted using the following program: 3 min of denaturation at 95 °C, 27 cycles of 30 s at 95 °C, 30 s for annealing at 55 °C, 45 s for elongations for 72 °C. and final extension at 72 °C for 10 min. PCR reactions were performed in a triplicate 20 µL mixture containing 4 µL of 5 × Fast Pfu Buffer, 2 µL of 2.5 mM dNTPs, of 0.8 µL of each primer (5 µM). 0.4 µL of FastPfu polymerase and 10 ng of template DNA. The resulted PCR products were extracted from a 2% agarose gel and further purified using the AxyPrep DNA Gel Extraction Kit (Axygen Biosciences, Union City, CA, USA) and quantified using QuantiFluo™-ST (Promega, USA), according to manufacture protocol.

### Bioinformatics and illumina MiSeq sequencing analysis

#### Illumina MiSeq sequencing

Purified amplicons were pooled in equimolar and paired-end sequenced (2 × 300) on an illumina MiSeq platform (Illumina, San Diego, USA) according to the standard protocols by Majorbio Bio-Pharm Technology Co. Ltd. (Shanghai, China).

#### Processing of sequencing data

Raw fastq files were demultiplexed, quality-filtered by Trimmomatic and merged by FLASH with the following criteria: (1) the reads were truncated at any site receiving an average quality score < 20 over a 50 bp sliding window. (2) Primers were exactly matched allowing two nucleotide mismatching, and reads containing ambiguous bases were removed. (3) Sequences whose overlap longer than10 bp were merged according to their overlap sequence^38^.

#### OTU cluster and taxonomy

Operational taxonomic units (OTUs) were clustered with 97% similarity cutoff using UPA (version 7.1 http://drive5.com/uparse/) and chimeric sequences were identified and removed using UCHIME. The taxonomy of each 16S rRNA gene sequence was analyzed by RDP Classifier algorithm (http://rdp.cme.msu.edu/) against the SILVA (SSU123) 16S rRNA database using confidence threshold of 70%. Phylogenetic analysis of the representative sequences was performed for aligned unique sequences of 16S rRNA using sanger software (i.sanger.com)^38^.

### Alpha diversity, beta diversity and rarefaction curve

A random sampling way was applied for the trimmed sequences and numbers of these relevant representative OTUs extracted sequences were established by the rarefaction curves. OTUs (97%) similarity data, alpha diversity was evaluated calculating the richness estimator (ACE estimator and Chao estimator show community affluent), the diversity indices (Simpson and Shannon index show community diversity including evenness and affluent), coverage shows sequence depth and sobs shows the total number of species noticed in each soil samples. Beta diversity was used for biological distance different samples species were calculated by Bray–Curtis distance^52,53^.

### Statistical analysis

While, working with single crop monoculture and interplant as a source of variable one-way ANOVA tests SPSS version 16.0 software and least significant difference (LSD) was applied to test for significance among the means. The co-relations between monoculture and intercropping and microbiological diversity parameters were analyzed using Pearson correlation analysis (two-tailed)^54^.

## Supplementary Information

Supplementary Figure S1.
