# Supplementary material for: Garlic (Allium sativum) based interplanting alters the heavy metals absorption and bacterial diversity in neighboring plants
Source: Sci Rep. 2021 Mar 12;11:5833. doi: 10.1038/s41598-021-85269-4 (PMC7971001; doi:10.1038/s41598-021-85269-4)
Supplement: Supplementary file 1 — Supplementary Figure S1. [file 41598_2021_85269_MOESM1_ESM.pdf]

Supplementary figure for: Garlic (Allium sativum) based interplanting alters the heavy metals absorption and bacterial diversity in neighboring plants

JAVED HUSSAIN[1*], XIAO WEI[1], LUO XUE-GANG[1], SYED REHMAT ULLAH SHAH[2], ALI MURAD JAKHAR[1], MUHAMMAD ASLAM[2] SHAIHK ABDULLAH[2] , ASMA BABAR[2],TOUQIR AZAM[1]

[1]College of Life Science and Engineering, Southwest University of Science and Technology, Mianyang Sichuan 621010 China

[2]Lasbela University of Agriculture, Water and Marine Sciences 90150 Uthal, Pakistan

Supplementary Fig. S1

Heatmap and Hierarchical cluster analysis of OTUs for bacterial communities. Colour coding indicates the relative abundance of each OTU. Clustered based on complete linkage method were applied for samples communities. D1 represents single plant and T1 interplanting communities.

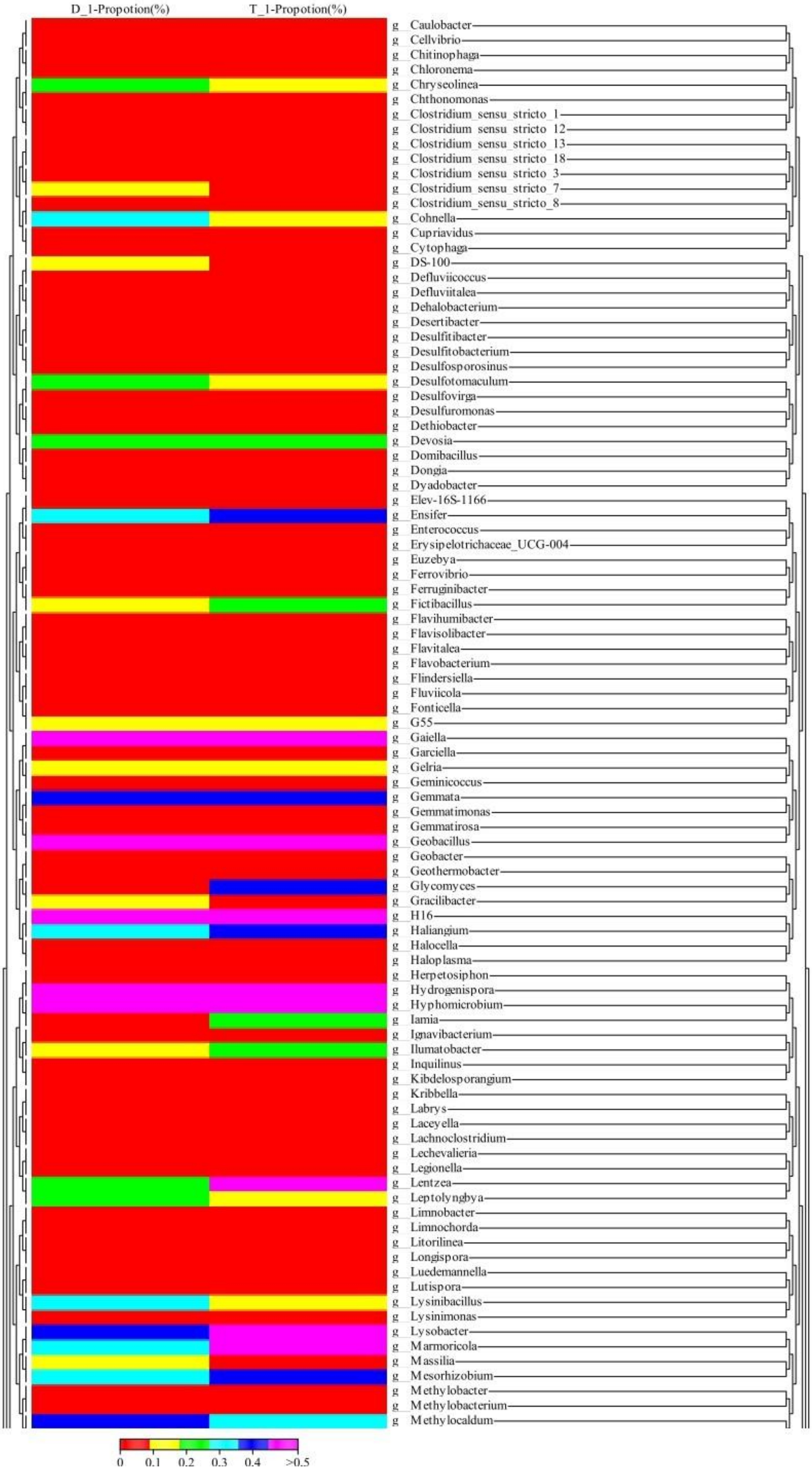
D_1-Propotion(%)
T_1-Propotion(%)
g__Caulobacter
g__Cellvibrio
g__Chitinophaga
g__Chloronema
g__Chryseolinea
g__Chthonomonas
g__Clostridium_sensu_stricto_1
g__Clostridium_sensu_stricto_12
g__Clostridium_sensu_stricto_13
g__Clostridium_sensu_stricto_18
g__Clostridium_sensu_stricto_3
g__Clostridium_sensu_stricto_7
g__Clostridium_sensu_stricto_8
g__Cohnella
g__Cupriavidus
g__Cytophaga
g__DS-100
g__Defluviicoccus
g__Defluviitalea
g__Dehalobacterium
g__Desertibacter
g__Desulfitibacter
g__Desulfitobacterium
g__Desulfosporosinus
g__Desulfotomaculum
g__Desulfovirga
g__Desulfuromonas
g__Dethiobacter
g__Devosia
g__Domibacillus
g__Dongia
g__Dyadobacter
g__Elev-16S-1166
g__Ensifer
g__Enterococcus
g__Erysipelotrichaceae_UCG-004
g__Euzebya
g__Ferrovibrio
g__Ferruginibacter
g__Fictibacillus
g__Flavihumibacter
g__Flavisolibacter
g__Flavitalea
g__Flavobacterium
g__Flindersiella
g__Fluviicola
g__Fonticella
g__G55
g__Gaiella
g__Garciella
g__Gelria
g__Geminicoccus
g__Gemmata
g__Gemmatimonas
g__Gemmatirosa
g__Geobacillus
g__Geobacter
g__Geothermobacter
g__Glycomyces
g__Gracilibacter
g__H16
g__Haliangium
g__Halocella
g__Haloplasma
g__Herpetosiphon
g__Hydrogenispora
g__Hyphomicrobium
g__Iamia
g__Ignavibacterium
g__Ilumatobacter
g__Inquilinus
g__Kibdelosporangium
g__Kribbella
g__Labrys
g__Laceyella
g__Lachnoclostridium
g__Lechevalieria
g__Legionella
g__Lentzea
g__Leptolyngbya
g__Limnobacter
g__Limnochorda
g__Litorilinea
g__Longispora
g__Luedemannella
g__Lutispora
g__Lysinibacillus
g__Lysinimonas
g__Lysobacter
g__Marmoricola
g__Massilia
g__Mesorhizobium
g__Methylobacter
g__Methylobacterium
g__Methylocaldum
0
0.1
0.2
0.3
0.4
>0.5

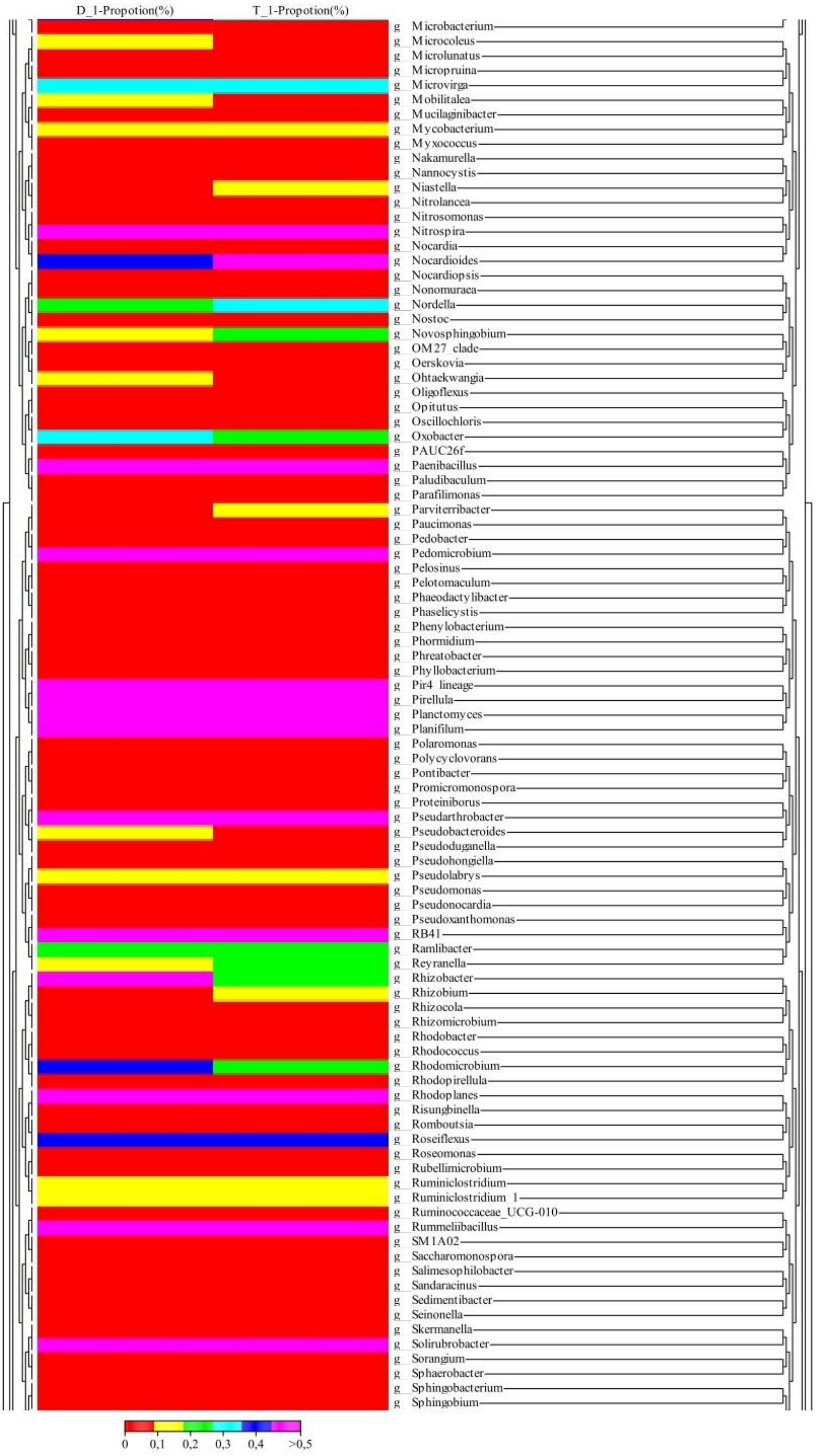
D_1-Propotion(%)
T_1-Propotion(%)
g_Microbacterium
g_Microcoleus
g_Microlunatus
g_Micropruina
g_Microvirga
g_Mobilitalea
g_Mucilaginibacter
g_Mycobacterium
g_Myxococcus
g_Nakamurella
g_Nannocystis
g_Niastella
g_Nitrolancea
g_Nitrosomonas
g_Nitrospira
g_Nocardia
g_Nocardioides
g_Nocardiopsis
g_Nonomuraea
g_Nordella
g_Nostoc
g_Novosphingobium
g_OM27_clade
g_Oerskovia
g_Ohtaekwangia
g_Oligoflexus
g_Opitutus
g_Oscillochloris
g_Oxobacter
g_PAUC26f
g_Paenibacillus
g_Paludibaculum
g_Parafilimonas
g_Parviterribacter
g_Paucimonas
g_Pedobacter
g_Pedomicrobium
g_Pelosinus
g_Pelotomaculum
g_Phaeodactylibacter
g_Phaselicystis
g_Phenylobacterium
g_Phormidium
g_Phreatobacter
g_Phyllobacterium
g_Pir4_lineage
g_Pirellula
g_Planctomyces
g_Planifilum
g_Polaromonas
g_Polycyclovorans
g_Pontibacter
g_Promicromonospora
g_Proteiniborus
g_Pseudarthrobacter
g_Pseudobacteroides
g_Pseudoduganella
g_Pseudohongiella
g_Pseudolabrys
g_Pseudomonas
g_Pseudonocardia
g_Pseudoxanthomonas
g_RB41
g_Ramlibacter
g_Reyranella
g_Rhizobacter
g_Rhizobium
g_Rhizocola
g_Rhizomicrobium
g_Rhodobacter
g_Rhodococcus
g_Rhodomicrobium
g_Rhodopirellula
g_Rhodoplanes
g_Risungbinella
g_Romboutsia
g_Roseiflexus
g_Roseomonas
g_Rubellimicrobium
g_Ruminiclostridium
g_Ruminiclostridium_1
g_Ruminococcaceae_UCG-010
g_Rummeliibacillus
g_SM1A02
g_Saccharomonospora
g_Salimesophilobacter
g_Sandaracinus
g_Sedimentibacter
g_Seinonella
g_Skermanella
g_Solirubrobacter
g_Sorangium
g_Sphaerobacter
g_Sphingobacterium
g_Sphingobium
0
0,1
0,2
0,3
0,4
>0,5

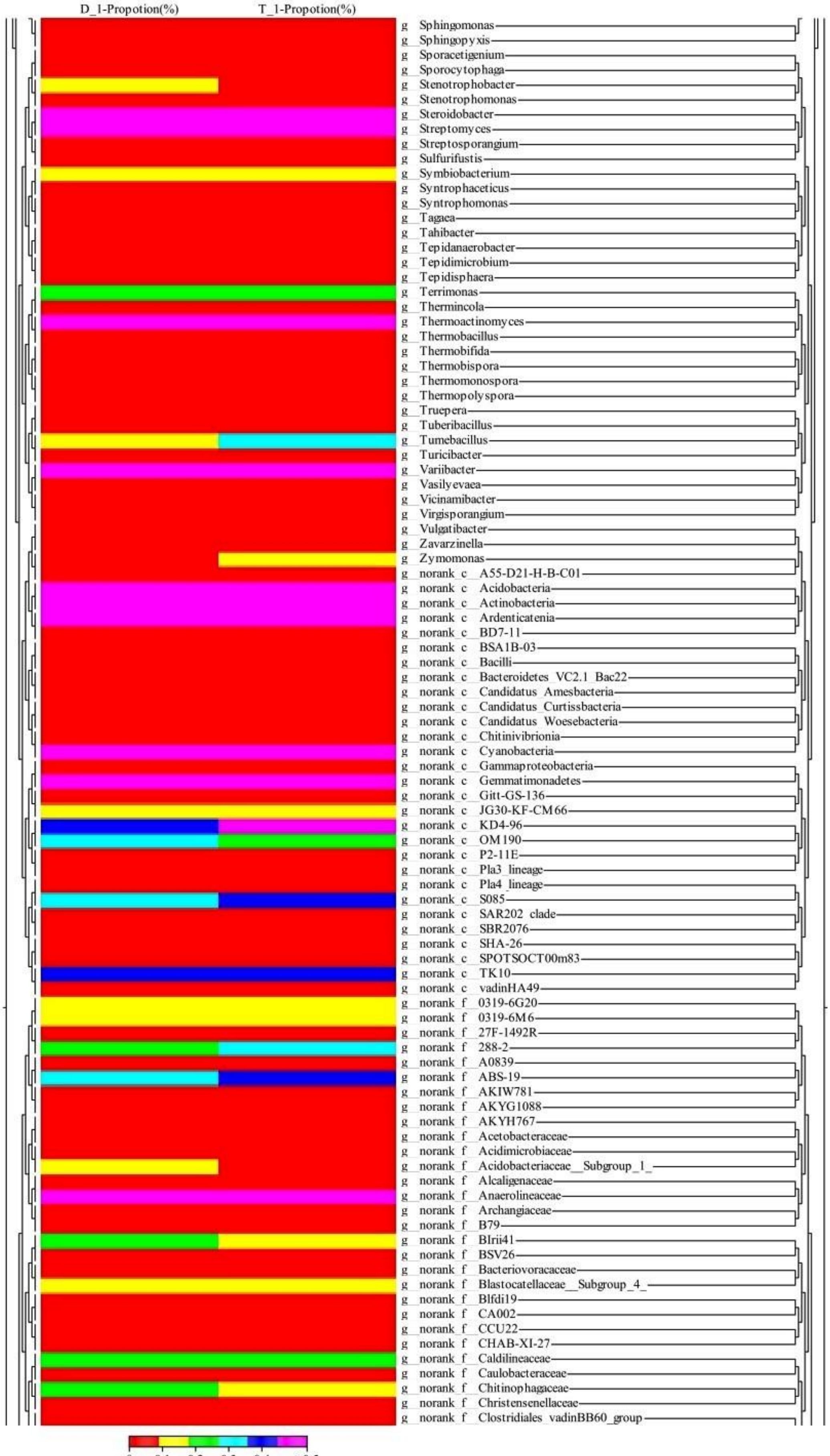

D_1-Propotion(%)
T_1-Propotion(%)
g__Sphingomonas
g__Sphingopyxis
g__Sporacetigenium
g__Sporocytophaga
g__Stenotrophobacter
g__Stenotrophomonas
g__Steroidobacter
g__Streptomyces
g__Streptosporangium
g__Sulfurifustis
g__Symbiobacterium
g__Syntrophaceticus
g__Syntrophomonas
g__Tagaea
g__Tahibacter
g__Tepidanaerobacter
g__Tepidimicrobium
g__Tepidisphaera
g__Terrimonas
g__Thermincola
g__Thermoactinomyces
g__Thermobacillus
g__Thermobifida
g__Thermobispora
g__Thermomonospora
g__Thermopolyspora
g__Truepera
g__Tuberibacillus
g__Tumebacillus
g__Turicibacter
g__Variibacter
g__Vasilyevaea
g__Vicinamibacter
g__Virgisporangium
g__Vulgatibacter
g__Zavarzinella
g__Zymomonas
g__norank_c__A55-D21-H-B-C01
g__norank_c__Acidobacteria
g__norank_c__Actinobacteria
g__norank_c__Ardenticatenia
g__norank_c__BD7-11
g__norank_c__BSA1B-03
g__norank_c__Bacilli
g__norank_c__Bacteroidetes_VC2.1_Bac22
g__norank_c__Candidatus_Amesbacteria
g__norank_c__Candidatus_Curtissbacteria
g__norank_c__Candidatus_Woesebacteria
g__norank_c__Chitinivibrionia
g__norank_c__Cyanobacteria
g__norank_c__Gammaproteobacteria
g__norank_c__Gemmatimonadetes
g__norank_c__Gitt-GS-136
g__norank_c__JG30-KF-CM66
g__norank_c__KD4-96
g__norank_c__OM190
g__norank_c__P2-11E
g__norank_c__Pla3_lineage
g__norank_c__Pla4_lineage
g__norank_c__S085
g__norank_c__SAR202_clade
g__norank_c__SBR2076
g__norank_c__SHA-26
g__norank_c__SPOTSOCT00m83
g__norank_c__TK10
g__norank_c__vadinHA49
g__norank_f__0319-6G20
g__norank_f__0319-6M6
g__norank_f__27F-1492R
g__norank_f__288-2
g__norank_f__A0839
g__norank_f__ABS-19
g__norank_f__AKIW781
g__norank_f__AKYG1088
g__norank_f__AKYH767
g__norank_f__Acetobacteraceae
g__norank_f__Acidimicrobiaceae
g__norank_f__Acidobacteriaceae__Subgroup_1_
g__norank_f__Alcaligenaceae
g__norank_f__Anaerolineaceae
g__norank_f__Archangiaceae
g__norank_f__B79
g__norank_f__BIrii41
g__norank_f__BSV26
g__norank_f__Bacteriovoracaceae
g__norank_f__Blastocatellaceae__Subgroup_4_
g__norank_f__Blfdi19
g__norank_f__CA002
g__norank_f__CCU22
g__norank_f__CHAB-XI-27
g__norank_f__Caldilineaceae
g__norank_f__Caulobacteraceae
g__norank_f__Chitinophagaceae
g__norank_f__Christensenellaceae
g__norank_f__Clostridiales_vadinBB60_group
0
0,1
0,2
0,3
0,4
>0,5

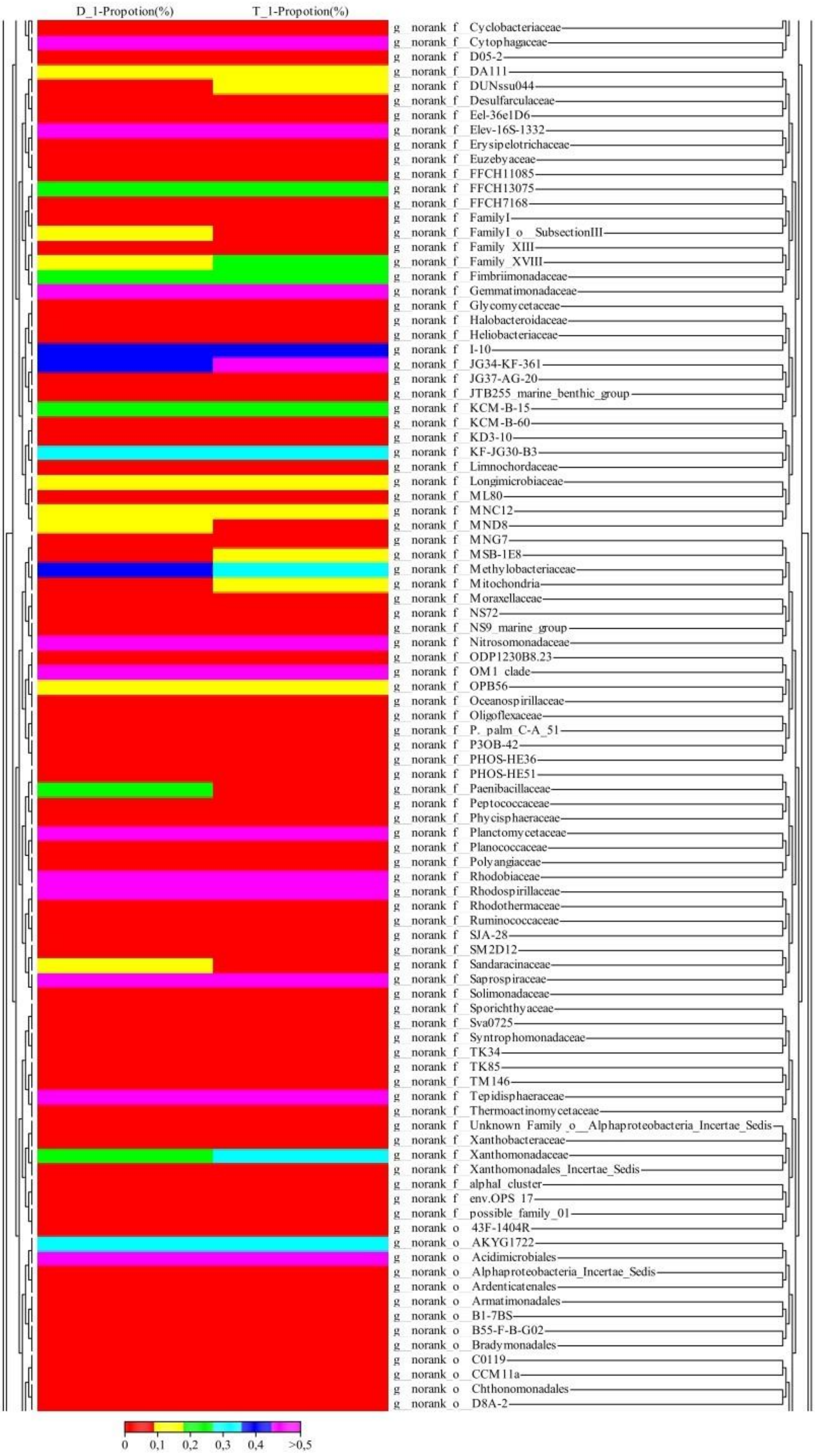

D_1-Propotion(%)
T_1-Propotion(%)
g norank f Cyclobacteriaceae
g norank f Cytophagaceae
g norank f D05-2
g norank f DA111
g norank f DUNssu044
g norank f Desulfarculaceae
g norank f Eel-36e1D6
g norank f Elev-16S-1332
g norank f Erysipelotrichaceae
g norank f Euzebyaceae
g norank f FFCH11085
g norank f FFCH13075
g norank f FFCH7168
g norank f FamilyI
g norank f FamilyI o SubsectionIII
g norank f Family XIII
g norank f Family XVIII
g norank f Fimbriimonadaceae
g norank f Gemmatimonadaceae
g norank f Glycomycetaceae
g norank f Halobacteroidaceae
g norank f Heliobacteriaceae
g norank f I-10
g norank f JG34-KF-361
g norank f JG37-AG-20
g norank f JTB255_marine_benthic_group
g norank f KCM-B-15
g norank f KCM-B-60
g norank f KD3-10
g norank f KF-JG30-B3
g norank f Limnochordaceae
g norank f Longimicrobiaceae
g norank f ML80
g norank f MNC12
g norank f MND8
g norank f MNG7
g norank f MSB-1E8
g norank f Methylobacteriaceae
g norank f Mitochondria
g norank f Moraxellaceae
g norank f NS72
g norank f NS9 marine group
g norank f Nitrosomonadaceae
g norank f ODP1230B8.23
g norank f OM1 clade
g norank f OPB56
g norank f Oceanospirillaceae
g norank f Oligoflexaceae
g norank f P. palm C-A_51
g norank f P3OB-42
g norank f PHOS-HE36
g norank f PHOS-HE51
g norank f Paenibacillaceae
g norank f Peptococcaceae
g norank f Phycisphaeraceae
g norank f Planctomycetaceae
g norank f Planococcaceae
g norank f Polyangiaceae
g norank f Rhodobiaceae
g norank f Rhodospirillaceae
g norank f Rhodothermaceae
g norank f Ruminococcaceae
g norank f SJA-28
g norank f SM2D12
g norank f Sandaracinaceae
g norank f Saprospiraceae
g norank f Solimonadaceae
g norank f Sporichthyaceae
g norank f Sva0725
g norank f Syntrophomonadaceae
g norank f TK34
g norank f TK85
g norank f TM146
g norank f Tepidisphaeraceae
g norank f Thermoactinomycetaceae
g norank f Unknown Family o Alphaproteobacteria_Incertae_Sedis
g norank f Xanthobacteraceae
g norank f Xanthomonadaceae
g norank f Xanthomonadales_Incertae_Sedis
g norank f alphaI cluster
g norank f env.OPS 17
g norank f possible family_01
g norank o 43F-1404R
g norank o AKYG1722
g norank o Acidimicrobiales
g norank o Alphaproteobacteria_Incertae_Sedis
g norank o Ardenticatenales
g norank o Armatimonadales
g norank o B1-7BS
g norank o B55-F-B-G02
g norank o Bradymonadales
g norank o C0119
g norank o CCM11a
g norank o Chthonomonadales
g norank o D8A-2
0
0,1
0,2
0,3
0,4
>0,5

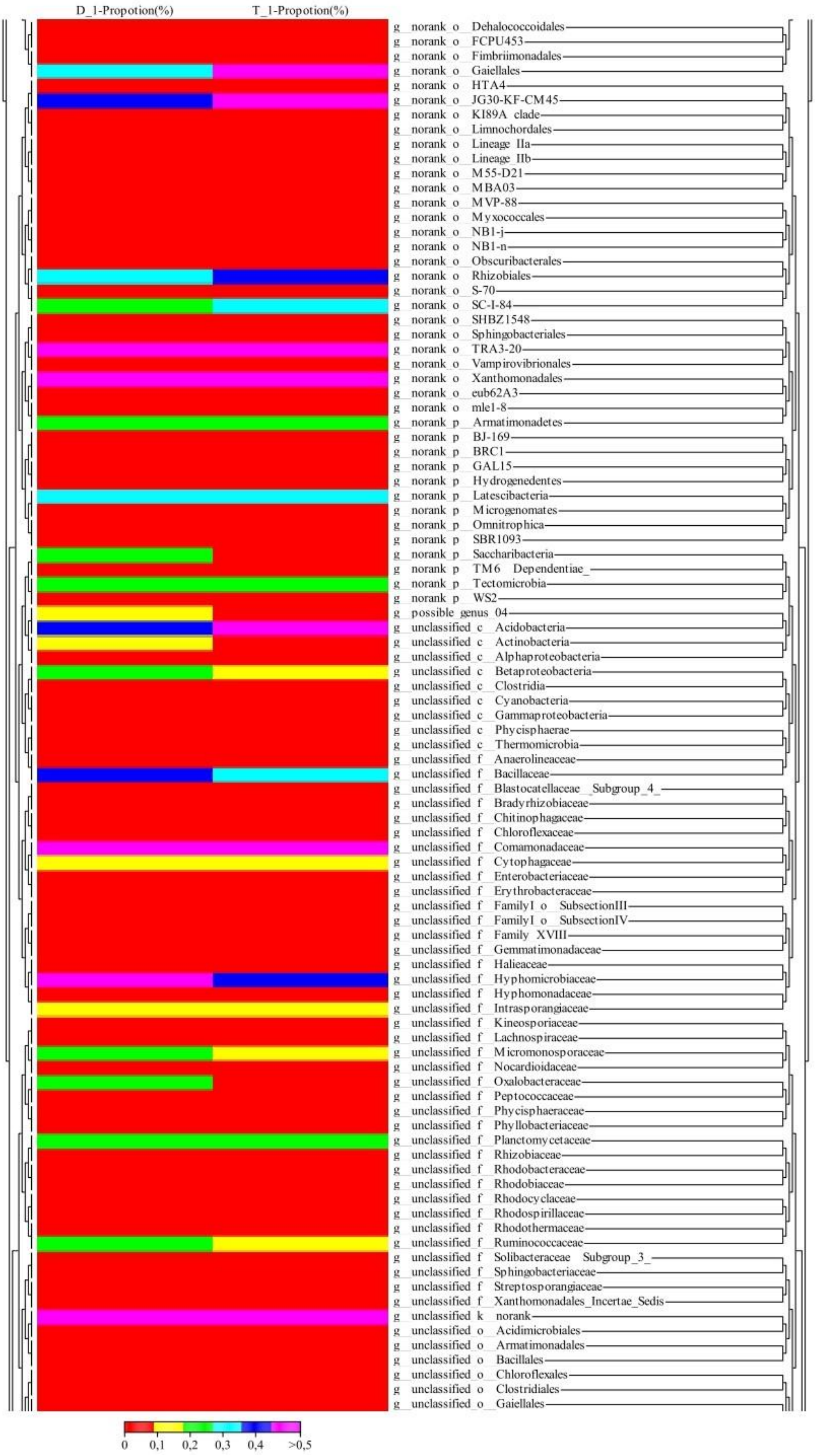
D_1-Propotion(%)
T_1-Propotion(%)
g norank o Dehalococcoidales
g norank o FCPU453
g norank o Fimbriimonadales
g norank o Gaiellales
g norank o HTA4
g norank o JG30-KF-CM45
g norank o KI89A clade
g norank o Limnochordales
g norank o Lineage IIa
g norank o Lineage IIb
g norank o M55-D21
g norank o MBA03
g norank o MVP-88
g norank o Myxococcales
g norank o NB1-j
g norank o NB1-n
g norank o Obscuribacterales
g norank o Rhizobiales
g norank o S-70
g norank o SC-I-84
g norank o SHBZ1548
g norank o Sphingobacteriales
g norank o TRA3-20
g norank o Vampirovibrionales
g norank o Xanthomonadales
g norank o eub62A3
g norank o mle1-8
g norank p Armatimonadetes
g norank p BJ-169
g norank p BRC1
g norank p GAL15
g norank p Hydrogenedentes
g norank p Latescibacteria
g norank p Microgenomates
g norank p Omnitrophica
g norank p SBR1093
g norank p Saccharibacteria
g norank p TM6 Dependentiae_
g norank p Tectomicrobia
g norank p WS2
g possible genus 04
g unclassified c Acidobacteria
g unclassified c Actinobacteria
g unclassified c Alphaproteobacteria
g unclassified c Betaproteobacteria
g unclassified c Clostridia
g unclassified c Cyanobacteria
g unclassified c Gammaproteobacteria
g unclassified c Phycisphaerae
g unclassified c Thermomicrobia
g unclassified f Anaerolineaceae
g unclassified f Bacillaceae
g unclassified f Blastocatellaceae Subgroup_4_
g unclassified f Bradyrhizobiaceae
g unclassified f Chitinophagaceae
g unclassified f Chloroflexaceae
g unclassified f Comamonadaceae
g unclassified f Cytophagaceae
g unclassified f Enterobacteriaceae
g unclassified f Erythrobacteraceae
g unclassified f FamilyI o SubsectionIII
g unclassified f FamilyI o SubsectionIV
g unclassified f Family XVIII
g unclassified f Gemmatimonadaceae
g unclassified f Halieaceae
g unclassified f Hyphomicrobiaceae
g unclassified f Hyphomonadaceae
g unclassified f Intrasporangiaceae
g unclassified f Kineosporiaceae
g unclassified f Lachnospiraceae
g unclassified f Micromonosporaceae
g unclassified f Nocardioidaceae
g unclassified f Oxalobacteraceae
g unclassified f Peptococcaceae
g unclassified f Phycisphaeraceae
g unclassified f Phyllobacteriaceae
g unclassified f Planctomycetaceae
g unclassified f Rhizobiaceae
g unclassified f Rhodobacteraceae
g unclassified f Rhodobiaceae
g unclassified f Rhodocyclaceae
g unclassified f Rhodospirillaceae
g unclassified f Rhodothermaceae
g unclassified f Ruminococcaceae
g unclassified f Solibacteraceae Subgroup_3_
g unclassified f Sphingobacteriaceae
g unclassified f Streptosporangiaceae
g unclassified f Xanthomonadales_Incertae_Sedis
g unclassified k norank
g unclassified o Acidimicrobiales
g unclassified o Armatimonadales
g unclassified o Bacillales
g unclassified o Chloroflexales
g unclassified o Clostridiales
g unclassified o Gaiellales
0
0,1
0,2
0,3
0,4
>0,5

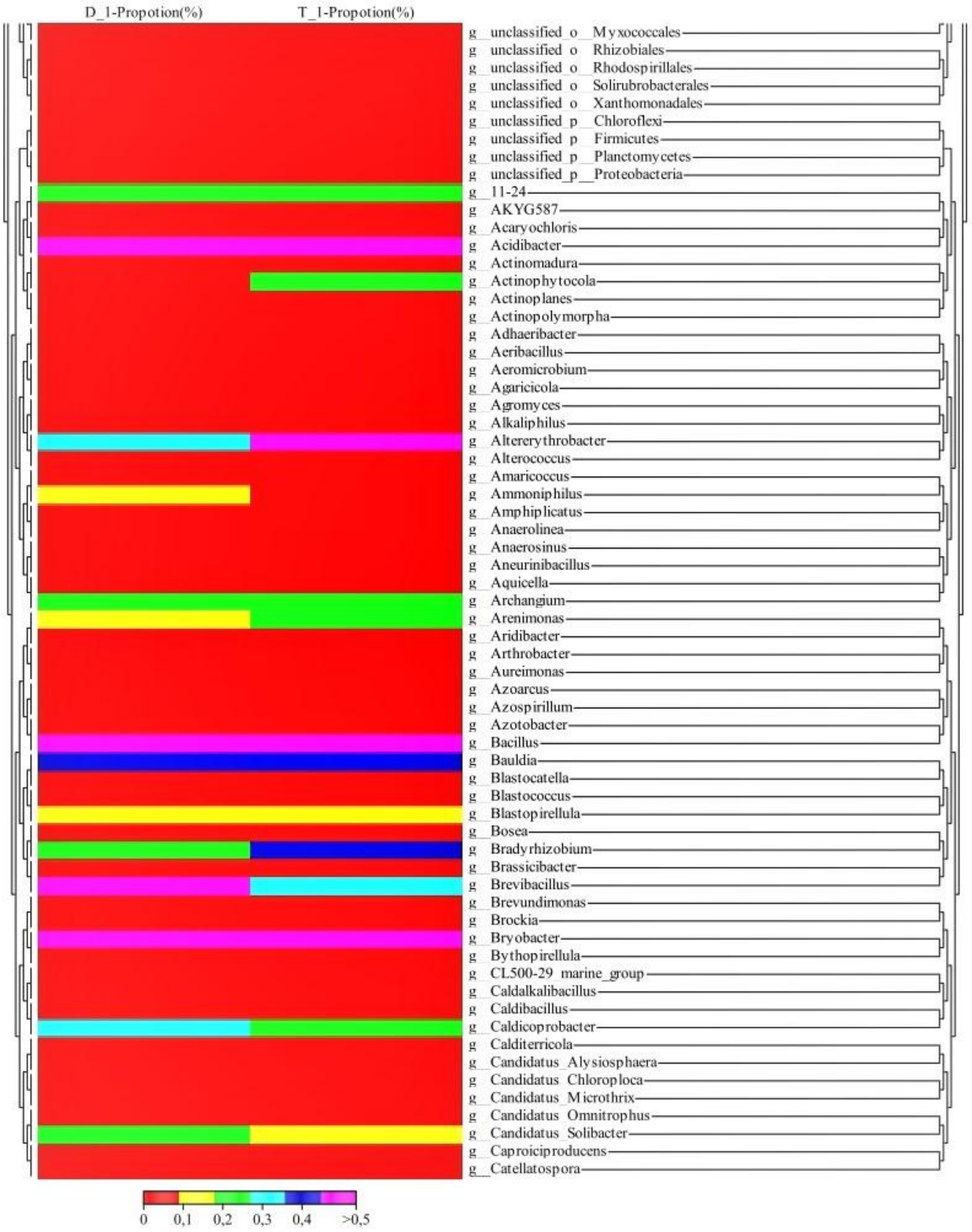

D_1-Propotion(%)
T_1-Propotion(%)
g__unclassified_o__Myxococcales
g__unclassified_o__Rhizobiales
g__unclassified_o__Rhodospirillales
g__unclassified_o__Solirubrobacterales
g__unclassified_o__Xanthomonadales
g__unclassified_p__Chloroflexi
g__unclassified_p__Firmicutes
g__unclassified_p__Planctomycetes
g__unclassified_p__Proteobacteria
g__11-24
g__AKYG587
g__Acaryochloris
g__Acidibacter
g__Actinomadura
g__Actinophytocola
g__Actinoplanes
g__Actinopolymorpha
g__Adhaeribacter
g__Aeribacillus
g__Aeromicrobium
g__Agaricicola
g__Agromyces
g__Alkaliphilus
g__Altererythrobacter
g__Alterococcus
g__Amaricoccus
g__Ammoniphilus
g__Amphiplicatus
g__Anaerolinea
g__Anaerosinus
g__Aneurinibacillus
g__Aquicella
g__Archangium
g__Arenimonas
g__Aridibacter
g__Arthrobacter
g__Aureimonas
g__Azoarcus
g__Azospirillum
g__Azotobacter
g__Bacillus
g__Bauldia
g__Blastocatella
g__Blastococcus
g__Blastopirellula
g__Bosea
g__Bradyrhizobium
g__Brassicibacter
g__Brevibacillus
g__Brevundimonas
g__Brockia
g__Bryobacter
g__Bythopirellula
g__CL500-29_marine_group
g__Caldalkalibacillus
g__Caldibacillus
g__Caldicoprobacter
g__Calditerricola
g__Candidatus_Alysiosphaera
g__Candidatus_Chloroploca
g__Candidatus_Microthrix
g__Candidatus_Omnitrophus
g__Candidatus_Solibacter
g__Caproiciproducens
g__Catellatospora
0
0,1
0,2
0,3
0,4
>0,5
